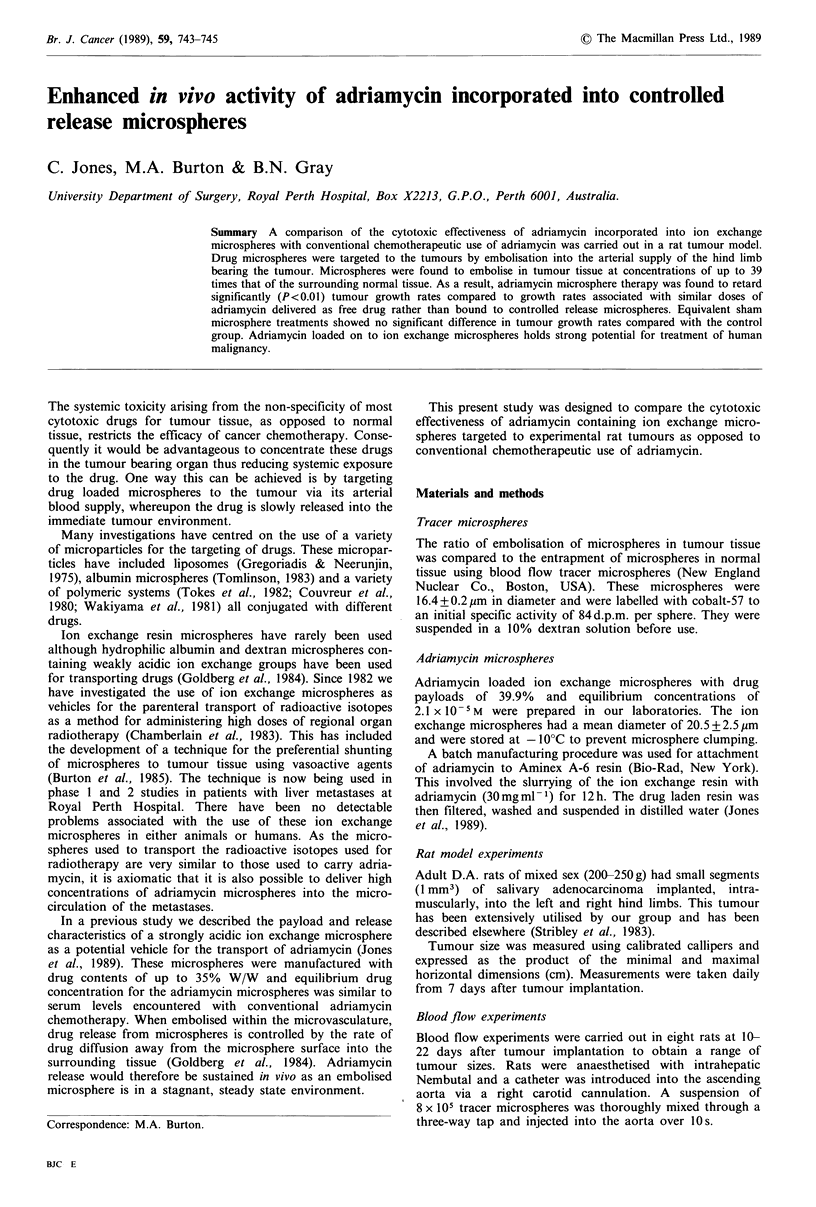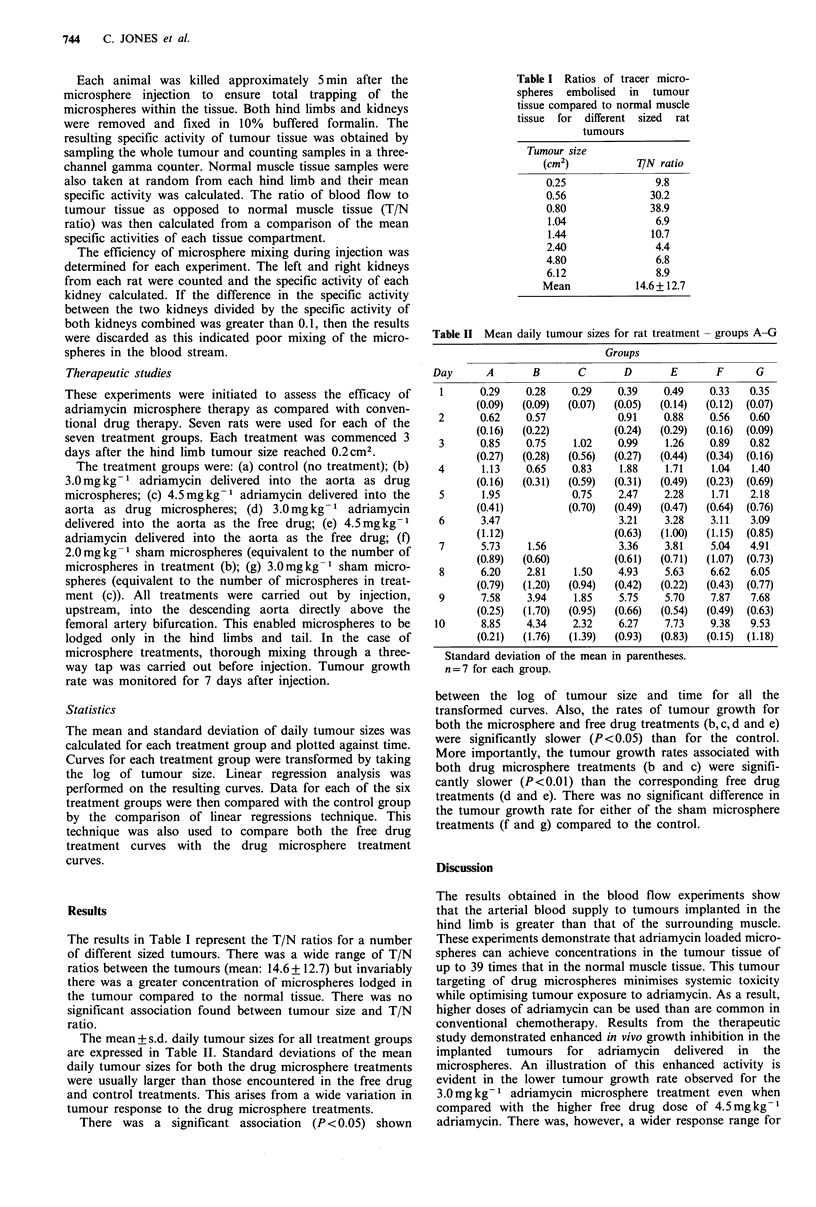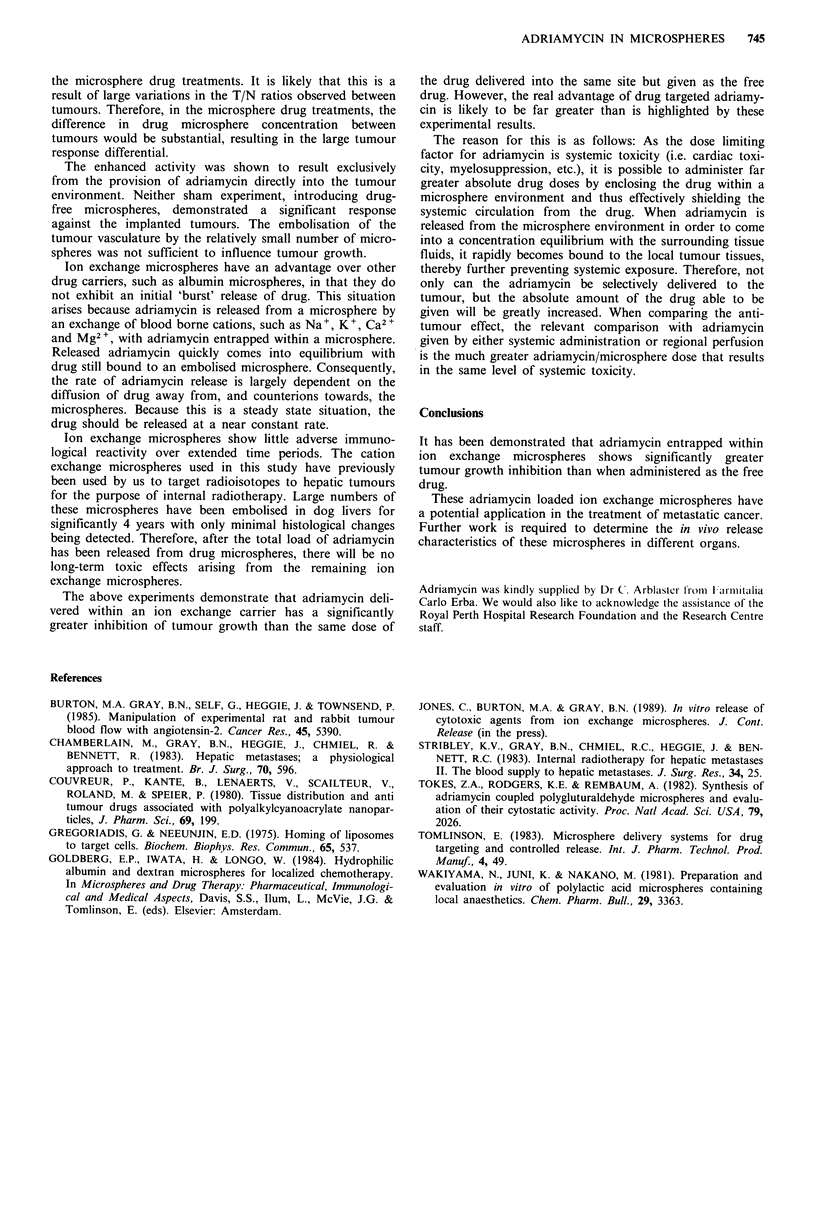# Enhanced in vivo activity of adriamycin incorporated into controlled release microspheres.

**DOI:** 10.1038/bjc.1989.155

**Published:** 1989-05

**Authors:** C. Jones, M. A. Burton, B. N. Gray

**Affiliations:** University Department of Surgery, Royal Perth Hospital, Australia.

## Abstract

A comparison of the cytotoxic effectiveness of adriamycin incorporated into ion exchange microspheres with conventional chemotherapeutic use of adriamycin was carried out in a rat tumour model. Drug microspheres were targeted to the tumours by embolisation into the arterial supply of the hind limb bearing the tumour. Microspheres were found to embolise in tumour tissue at concentrations of up to 39 times that of the surrounding normal tissue. As a result, adriamycin microsphere therapy was found to retard significantly (P less than 0.01) tumour growth rates compared to growth rates associated with similar doses of adriamycin delivered as free drug rather than bound to controlled release microspheres. Equivalent sham microsphere treatments showed no significant difference in tumour growth rates compared with the control group. Adriamycin loaded on to ion exchange microspheres holds strong potential for treatment of human malignancy.


Br. J. Cancer (1989), 59, 743–745 © The Macmillan Press Ltd., 1989

### Enhanced *in vivo* activity of adriamycin incorporated into controlled release microspheres

C. Jones, M.A. Burton & B.N. Gray

*University Department of Surgery, Royal Perth Hospital, Box X2213, G.P.O., Perth 6001, Australia.*

**Summary** A comparison of the cytotoxic effectiveness of adriamycin incorporated into ion exchange microspheres with conventional chemotherapeutic use of adriamycin was carried out in a rat tumour model. Drug microspheres were targeted to the tumours by embolisation into the arterial supply of the hind limb bearing the tumour. Microspheres were found to embolise in tumour tissue at concentrations of up to 39 times that of the surrounding normal tissue. As a result, adriamycin microsphere therapy was found to retard significantly ($P<0.01$) tumour growth rates compared to growth rates associated with similar doses of adriamycin delivered as free drug rather than bound to controlled release microspheres. Equivalent sham microsphere treatments showed no significant difference in tumour growth rates compared with the control group. Adriamycin loaded on to ion exchange microspheres holds strong potential for treatment of human malignancy.

The systemic toxicity arising from the non-specificity of most cytotoxic drugs for tumour tissue, as opposed to normal tissue, restricts the efficacy of cancer chemotherapy. Consequently it would be advantageous to concentrate these drugs in the tumour bearing organ thus reducing systemic exposure to the drug. One way this can be achieved is by targeting drug loaded microspheres to the tumour via its arterial blood supply, whereupon the drug is slowly released into the immediate tumour environment.

Many investigations have centred on the use of a variety of microparticles for the targeting of drugs. These microparticles have included liposomes (Gregoriadis & Neerunjin, 1975), albumin microspheres (Tomlinson, 1983) and a variety of polymeric systems (Tokes *et al.*, 1982; Couvreur *et al.*, 1980; Wakiyama *et al.*, 1981) all conjugated with different drugs.

Ion exchange resin microspheres have rarely been used although hydrophilic albumin and dextran microspheres containing weakly acidic ion exchange groups have been used for transporting drugs (Goldberg *et al.*, 1984). Since 1982 we have investigated the use of ion exchange microspheres as vehicles for the parenteral transport of radioactive isotopes as a method for administering high doses of regional organ radiotherapy (Chamberlain *et al.*, 1983). This has included the development of a technique for the preferential shunting of microspheres to tumour tissue using vasoactive agents (Burton *et al.*, 1985). The technique is now being used in phase 1 and 2 studies in patients with liver metastases at Royal Perth Hospital. There have been no detectable problems associated with the use of these ion exchange microspheres in either animals or humans. As the microspheres used to transport the radioactive isotopes used for radiotherapy are very similar to those used to carry adriamycin, it is axiomatic that it is also possible to deliver high concentrations of adriamycin microspheres into the microcirculation of the metastases.

In a previous study we described the payload and release characteristics of a strongly acidic ion exchange microsphere as a potential vehicle for the transport of adriamycin (Jones *et al.*, 1989). These microspheres were manufactured with drug contents of up to 35% W/W and equilibrium drug concentration for the adriamycin microspheres was similar to serum levels encountered with conventional adriamycin chemotherapy. When embolised within the microvasculature, drug release from microspheres is controlled by the rate of drug diffusion away from the microsphere surface into the surrounding tissue (Goldberg *et al.*, 1984). Adriamycin release would therefore be sustained *in vivo* as an embolised microsphere is in a stagnant, steady state environment.

Correspondence: M.A. Burton.

This present study was designed to compare the cytotoxic effectiveness of adriamycin containing ion exchange microspheres targeted to experimental rat tumours as opposed to conventional chemotherapeutic use of adriamycin.

### Materials and methods

### *Tracer microspheres*

The ratio of embolisation of microspheres in tumour tissue was compared to the entrapment of microspheres in normal tissue using blood flow tracer microspheres (New England Nuclear Co., Boston, USA). These microspheres were $16.4 \pm 0.2\,\mu m$ in diameter and were labelled with cobalt-57 to an initial specific activity of 84 d.p.m. per sphere. They were suspended in a 10% dextran solution before use.

### *Adriamycin microspheres*

Adriamycin loaded ion exchange microspheres with drug payloads of 39.9% and equilibrium concentrations of $2.1 \times 10^{-5}$ M were prepared in our laboratories. The ion exchange microspheres had a mean diameter of $20.5 \pm 2.5\,\mu m$ and were stored at $-10°C$ to prevent microsphere clumping.

A batch manufacturing procedure was used for attachment of adriamycin to Aminex A-6 resin (Bio-Rad, New York). This involved the slurrying of the ion exchange resin with adriamycin ($30\,mg\,ml^{-1}$) for 12 h. The drug laden resin was then filtered, washed and suspended in distilled water (Jones *et al.*, 1989).

### *Rat model experiments*

Adult D.A. rats of mixed sex (200–250 g) had small segments ($1\,mm^3$) of salivary adenocarcinoma implanted, intramuscularly, into the left and right hind limbs. This tumour has been extensively utilised by our group and has been described elsewhere (Stribley *et al.*, 1983).

Tumour size was measured using calibrated callipers and expressed as the product of the minimal and maximal horizontal dimensions (cm). Measurements were taken daily from 7 days after tumour implantation.

### *Blood flow experiments*

Blood flow experiments were carried out in eight rats at 10–22 days after tumour implantation to obtain a range of tumour sizes. Rats were anaesthetised with intrahepatic Nembutal and a catheter was introduced into the ascending aorta via a right carotid cannulation. A suspension of $8 \times 10^5$ tracer microspheres was thoroughly mixed through a three-way tap and injected into the aorta over 10 s.

Each animal was killed approximately 5 min after the microsphere injection to ensure total trapping of the microspheres within the tissue. Both hind limbs and kidneys were removed and fixed in 10% buffered formalin. The resulting specific activity of tumour tissue was obtained by sampling the whole tumour and counting samples in a three-channel gamma counter. Normal muscle tissue samples were also taken at random from each hind limb and their mean specific activity was calculated. The ratio of blood flow to tumour tissue as opposed to normal muscle tissue (T/N ratio) was then calculated from a comparison of the mean specific activities of each tissue compartment.

The efficiency of microsphere mixing during injection was determined for each experiment. The left and right kidneys from each rat were counted and the specific activity of each kidney calculated. If the difference in the specific activity between the two kidneys divided by the specific activity of both kidneys combined was greater than 0.1, then the results were discarded as this indicated poor mixing of the microspheres in the blood stream.

*Therapeutic studies*

These experiments were initiated to assess the efficacy of adriamycin microsphere therapy as compared with conventional drug therapy. Seven rats were used for each of the seven treatment groups. Each treatment was commenced 3 days after the hind limb tumour size reached 0.2 $cm^2$.

The treatment groups were: (a) control (no treatment); (b) 3.0 $mg\,kg^{-1}$ adriamycin delivered into the aorta as drug microspheres; (c) 4.5 $mg\,kg^{-1}$ adriamycin delivered into the aorta as drug microspheres; (d) 3.0 $mg\,kg^{-1}$ adriamycin delivered into the aorta as the free drug; (e) 4.5 $mg\,kg^{-1}$ adriamycin delivered into the aorta as the free drug; (f) 2.0 $mg\,kg^{-1}$ sham microspheres (equivalent to the number of microspheres in treatment (b); (g) 3.0 $mg\,kg^{-1}$ sham microspheres (equivalent to the number of microspheres in treatment (c)). All treatments were carried out by injection, upstream, into the descending aorta directly above the femoral artery bifurcation. This enabled microspheres to be lodged only in the hind limbs and tail. In the case of microsphere treatments, thorough mixing through a three-way tap was carried out before injection. Tumour growth rate was monitored for 7 days after injection.

*Statistics*

The mean and standard deviation of daily tumour sizes was calculated for each treatment group and plotted against time. Curves for each treatment group were transformed by taking the log of tumour size. Linear regression analysis was performed on the resulting curves. Data for each of the six treatment groups were then compared with the control group by the comparison of linear regressions technique. This technique was also used to compare both the free drug treatment curves with the drug microsphere treatment curves.

### Results

The results in Table I represent the T/N ratios for a number of different sized tumours. There was a wide range of T/N ratios between the tumours (mean: $14.6 \pm 12.7$) but invariably there was a greater concentration of microspheres lodged in the tumour compared to the normal tissue. There was no significant association found between tumour size and T/N ratio.

The mean $\pm$ s.d. daily tumour sizes for all treatment groups are expressed in Table II. Standard deviations of the mean daily tumour sizes for both the drug microsphere treatments were usually larger than those encountered in the free drug and control treatments. This arises from a wide variation in tumour response to the drug microsphere treatments.

There was a significant association ($P<0.05$) shown between the log of tumour size and time for all the transformed curves. Also, the rates of tumour growth for both the microsphere and free drug treatments (b, c, d and e) were significantly slower ($P<0.05$) than for the control. More importantly, the tumour growth rates associated with both drug microsphere treatments (b and c) were significantly slower ($P<0.01$) than the corresponding free drug treatments (d and e). There was no significant difference in the tumour growth rate for either of the sham microsphere treatments (f and g) compared to the control.

**Table I** Ratios of tracer microspheres embolised in tumour tissue compared to normal muscle tissue for different sized rat tumours

| *Tumour size* ($cm^2$) | *T/N ratio* |
|---|---|
| 0.25 | 9.8 |
| 0.56 | 30.2 |
| 0.80 | 38.9 |
| 1.04 | 6.9 |
| 1.44 | 10.7 |
| 2.40 | 4.4 |
| 4.80 | 6.8 |
| 6.12 | 8.9 |
| Mean | $14.6 \pm 12.7$ |

**Table II** Mean daily tumour sizes for rat treatment – groups A–G

| | *Groups* | | | | | | |
|---|---|---|---|---|---|---|---|
| *Day* | *A* | *B* | *C* | *D* | *E* | *F* | *G* |
| 1 | 0.29 | 0.28 | 0.29 | 0.39 | 0.49 | 0.33 | 0.35 |
| | (0.09) | (0.09) | (0.07) | (0.05) | (0.14) | (0.12) | (0.07) |
| 2 | 0.62 | 0.57 | | 0.91 | 0.88 | 0.56 | 0.60 |
| | (0.16) | (0.22) | | (0.24) | (0.29) | (0.16) | (0.09) |
| 3 | 0.85 | 0.75 | 1.02 | 0.99 | 1.26 | 0.89 | 0.82 |
| | (0.27) | (0.28) | (0.56) | (0.27) | (0.44) | (0.34) | (0.16) |
| 4 | 1.13 | 0.65 | 0.83 | 1.88 | 1.71 | 1.04 | 1.40 |
| | (0.16) | (0.31) | (0.59) | (0.31) | (0.49) | (0.23) | (0.69) |
| 5 | 1.95 | | 0.75 | 2.47 | 2.28 | 1.71 | 2.18 |
| | (0.41) | | (0.70) | (0.49) | (0.47) | (0.64) | (0.76) |
| 6 | 3.47 | | | 3.21 | 3.28 | 3.11 | 3.09 |
| | (1.12) | | | (0.63) | (1.00) | (1.15) | (0.85) |
| 7 | 5.73 | 1.56 | | 3.36 | 3.81 | 5.04 | 4.91 |
| | (0.89) | (0.60) | | (0.61) | (0.71) | (1.07) | (0.73) |
| 8 | 6.20 | 2.81 | 1.50 | 4.93 | 5.63 | 6.62 | 6.05 |
| | (0.79) | (1.20) | (0.94) | (0.42) | (0.22) | (0.43) | (0.77) |
| 9 | 7.58 | 3.94 | 1.85 | 5.75 | 5.70 | 7.87 | 7.68 |
| | (0.25) | (1.70) | (0.95) | (0.66) | (0.54) | (0.49) | (0.63) |
| 10 | 8.85 | 4.34 | 2.32 | 6.27 | 7.73 | 9.38 | 9.53 |
| | (0.21) | (1.76) | (1.39) | (0.93) | (0.83) | (0.15) | (1.18) |

Standard deviation of the mean in parentheses.
$n=7$ for each group.

### Discussion

The results obtained in the blood flow experiments show that the arterial blood supply to tumours implanted in the hind limb is greater than that of the surrounding muscle. These experiments demonstrate that adriamycin loaded microspheres can achieve concentrations in the tumour tissue of up to 39 times that in the normal muscle tissue. This tumour targeting of drug microspheres minimises systemic toxicity while optimising tumour exposure to adriamycin. As a result, higher doses of adriamycin can be used than are common in conventional chemotherapy. Results from the therapeutic study demonstrated enhanced *in vivo* growth inhibition in the implanted tumours for adriamycin delivered in the microspheres. An illustration of this enhanced activity is evident in the lower tumour growth rate observed for the 3.0 $mg\,kg^{-1}$ adriamycin microsphere treatment even when compared with the higher free drug dose of 4.5 $mg\,kg^{-1}$ adriamycin. There was, however, a wider response range for the microsphere drug treatments. It is likely that this is a result of large variations in the T/N ratios observed between tumours. Therefore, in the microsphere drug treatments, the difference in drug microsphere concentration between tumours would be substantial, resulting in the large tumour response differential.

The enhanced activity was shown to result exclusively from the provision of adriamycin directly into the tumour environment. Neither sham experiment, introducing drug-free microspheres, demonstrated a significant response against the implanted tumours. The embolisation of the tumour vasculature by the relatively small number of microspheres was not sufficient to influence tumour growth.

Ion exchange microspheres have an advantage over other drug carriers, such as albumin microspheres, in that they do not exhibit an initial 'burst' release of drug. This situation arises because adriamycin is released from a microsphere by an exchange of blood borne cations, such as $Na^+$, $K^+$, $Ca^{2+}$ and $Mg^{2+}$, with adriamycin entrapped within a microsphere. Released adriamycin quickly comes into equilibrium with drug still bound to an embolised microsphere. Consequently, the rate of adriamycin release is largely dependent on the diffusion of drug away from, and counterions towards, the microspheres. Because this is a steady state situation, the drug should be released at a near constant rate.

Ion exchange microspheres show little adverse immunological reactivity over extended time periods. The cation exchange microspheres used in this study have previously been used by us to target radioisotopes to hepatic tumours for the purpose of internal radiotherapy. Large numbers of these microspheres have been embolised in dog livers for significantly 4 years with only minimal histological changes being detected. Therefore, after the total load of adriamycin has been released from drug microspheres, there will be no long-term toxic effects arising from the remaining ion exchange microspheres.

The above experiments demonstrate that adriamycin delivered within an ion exchange carrier has a significantly greater inhibition of tumour growth than the same dose of the drug delivered into the same site but given as the free drug. However, the real advantage of drug targeted adriamycin is likely to be far greater than is highlighted by these experimental results.

The reason for this is as follows: As the dose limiting factor for adriamycin is systemic toxicity (i.e. cardiac toxicity, myelosuppression, etc.), it is possible to administer far greater absolute drug doses by enclosing the drug within a microsphere environment and thus effectively shielding the systemic circulation from the drug. When adriamycin is released from the microsphere environment in order to come into a concentration equilibrium with the surrounding tissue fluids, it rapidly becomes bound to the local tumour tissues, thereby further preventing systemic exposure. Therefore, not only can the adriamycin be selectively delivered to the tumour, but the absolute amount of the drug able to be given will be greatly increased. When comparing the anti-tumour effect, the relevant comparison with adriamycin given by either systemic administration or regional perfusion is the much greater adriamycin/microsphere dose that results in the same level of systemic toxicity.

### Conclusions

It has been demonstrated that adriamycin entrapped within ion exchange microspheres shows significantly greater tumour growth inhibition than when administered as the free drug.

These adriamycin loaded ion exchange microspheres have a potential application in the treatment of metastatic cancer. Further work is required to determine the *in vivo* release characteristics of these microspheres in different organs.

Adriamycin was kindly supplied by Dr C. Arblaster from Farmitalia Carlo Erba. We would also like to acknowledge the assistance of the Royal Perth Hospital Research Foundation and the Research Centre staff.

### References

BURTON, M.A. GRAY, B.N., SELF, G., HEGGIE, J. & TOWNSEND, P. (1985). Manipulation of experimental rat and rabbit tumour blood flow with angiotensin-2. *Cancer Res.*, **45**, 5390.

CHAMBERLAIN, M., GRAY, B.N., HEGGIE, J., CHMIEL, R. & BENNETT, R. (1983). Hepatic metastases; a physiological approach to treatment. *Br. J. Surg.*, **70**, 596.

COUVREUR, P., KANTE, B., LENAERTS, V., SCAILTEUR, V., ROLAND, M. & SPEIER, P. (1980). Tissue distribution and anti tumour drugs associated with polyalkylcyanoacrylate nanoparticles, *J. Pharm. Sci.*, **69**, 199.

GREGORIADIS, G. & NEEUNJIN, E.D. (1975). Homing of liposomes to target cells. *Biochem. Biophys. Res. Commun.*, **65**, 537.

GOLDBERG, E.P., IWATA, H. & LONGO, W. (1984). Hydrophilic albumin and dextran microspheres for localized chemotherapy. In *Microspheres and Drug Therapy: Pharmaceutical, Immunological and Medical Aspects*, Davis, S.S., Ilum, L., McVie, J.G. & Tomlinson, E. (eds). Elsevier: Amsterdam.

JONES, C., BURTON, M.A. & GRAY, B.N. (1989). *In vitro* release of cytotoxic agents from ion exchange microspheres. *J. Cont. Release* (in the press).

STRIBLEY, K.V., GRAY, B.N., CHMIEL, R.C., HEGGIE, J. & BENNETT, R.C. (1983). Internal radiotherapy for hepatic metastases II. The blood supply to hepatic metastases. *J. Surg. Res.*, **34**, 25.

TOKES, Z.A., RODGERS, K.E. & REMBAUM, A. (1982). Synthesis of adriamycin coupled polygluturaldehyde microspheres and evaluation of their cytostatic activity. *Proc. Natl Acad. Sci. USA*, **79**, 2026.

TOMLINSON, E. (1983). Microsphere delivery systems for drug targeting and controlled release. *Int. J. Pharm. Technol. Prod. Manuf.*, **4**, 49.

WAKIYAMA, N., JUNI, K. & NAKANO, M. (1981). Preparation and evaluation *in vitro* of polylactic acid microspheres containing local anaesthetics. *Chem. Pharm. Bull.*, **29**, 3363.